# Cyclosporine for the Treatment of HTLV-1-Induced HAM/TSP: An Experience from a Case Report

**DOI:** 10.1097/MD.0000000000000382

**Published:** 2015-01-09

**Authors:** Adrián Sánchez-Montalvá, Fernando Salvador, Estrella Caballero, Israel Molina

**Affiliations:** From the Infectious Diseases Department (AS-M, FS, IM), PROSICS (International Health Program of the Catalan Health Institute), Vall d’Hebron University Hospital, Universitat Autonòma de Barcelona, Barcelona, Spain; and Microbiology Department (EC), Vall d’Hebron Universitary Hospital, Universitat Autònoma de Barcelona, Barcelona, Spain.

## Abstract

HTLV-1-associated myelopathy/tropical spastic paraparesis (HAM/TSP) remains a challenging disease. Treatment options are scarce, and their safety and efficacy are currently a matter of concern.

We present a case report describing our experience using cyclosporine in a patient with early HAM/TSP who started with a gait disturbance at Vall d’Hebron University Hospital (Barcelona) from August 2012 to October 2013. After 62 weeks of treatment, clinical improvement was observed and proviral load diminished. No safety concerns were observed.

Cyclosporine seems to be effective in new-onset HAM/TSP or in chronic HAM/TSP that develops a relapse. However, the duration and safety profile of this steroid-sparing therapy remain unknown and should be further investigated.

## INTRODUCTION

Human T-lymphotropic virus (HTLV) was the first retrovirus to be described.^1^ Currently, 4 types of HTLV have been reported,^2^ with HTLV-1 being the most clinically relevant. Approximately, 20 million people are infected with HTLV-1 and 5 million with HTLV-2 worldwide.^3^ HTLV-1 infection is mainly present in the Sub-Saharan region, Japan, the Caribbean region, and some parts of Latin America, whereas HTLV-2 infection predominates in specific ethnic groups in Africa and America. Additionally, HTLV-2 infection has been found in injection drug users.^4^ Prevalence in the high endemic Japanese population has been rated at 0.66% in men and 1.02% in women.^5^ In Spain, the prevalence rate is 0.06% for HTLV-1 and 0.08% for HTLV-2. HTLV-1 was mainly diagnosed in the migrant population.^4^

The HTLV-1 infection has been associated with 2 life-threatening diseases, T-cell leukemia/lymphoma (TLL) and HTLV-1-associated myelopathy/tropical spastic paraparesis (HAM/TSP). Other manifestations, such as eye and skin involvement, have also been related with HTLV-1.^6,7^

HAM/TSP is an inflammatory disease of the central nervous system. The incidence of HAM/TSP was 5.3 cases per 1000 HTLV-1-seropositive cases per year in a study carried out in Brazil.^8^ Symptoms of the disease are insidious and difficult to interpret at the onset of the disease. Paraparesis, spasticity, urinary incontinence, lower back pain, and hyperreflexia are common but not specific findings, hindering both diagnosis and follow-up. A new classification to make monitoring of the therapeutic response easier was developed recently using onset, progression, and activity criteria.^9^

Although HAM/TSP has been known for decades and its consequences are devastating, treatment options are still scarce. Initially, antiretroviral therapy was used with contradictory results. Once the immune-mediated theory emerged, immunosuppressive therapy was studied. To date, cyclosporine and interferon-α are the preferred immunosuppressive agents, even though the evidence regarding their efficacy is limited.^9,10^

Host–virus interaction is crucial for the HTLV-1 spread and the development of lymphocyte-mediated related diseases. Immunomodulation with cyclosporine after HTLV-1 infection in a rabbit model has shown a decrease of proviral load.^11^ In a recent proof-of-concept study, 7 patients with HAM/TSP in an early/progressive stage of the disease were treated with cyclosporine with encouraging results.^10^

Based on the cyclosporine proof-of-concept study, we relate our experience with cyclosporine in a patient diagnosed with an early HAM/TSP. We utilized the same stage classification and outcome criteria as described by Martin et al.^10^

The institutional review board of the Vall d’Hebron University Hospital approved the study. The patient signed a written informed consent for publication of the case report.

## CASE

A 40-year-old woman from the Dominican Republic presented at the emergency room in August 2012 with an 8-month history of gait disturbances and falls. The patient had been living in Spain for the last 14 years and reported no travel history in the last 5 years. Her previous medical status unveiled a treated hyperthyroidism with a normal hormonal study in the follow up, a major depression disorder, and urinary bladder disturbances that had appeared during the previous year and were being studied at another center. She was under treatment with paroxetine and solifenacin succinate.

A more accurate medical history revealed blurred vision and muscle spasms. On physical examination on admission, the patient had normal axillary temperature, blood pressure values, and heart rate. Heart, lung, and abdominal examinations were unremarkable. Skin examination showed a pruriginous papular rash in the lower abdomen and scalp. Muscle strength was slightly reduced in the lower limbs, where the deep tendon reflexes were brisk and the reflex area expanded. Moreover, the lower limbs were spastic and vibratory sensation was diminished. The Babinski sign was presented bilaterally. On eye examination, uveitis was diagnosed.

In the blood test, the white cell count only showed a total eosinophil count of 1000 cells/mm^3^ (10.2%). Biochemical parameters remained within the normal range. Serological tests for hepatitis B and C, human immunodeficiency virus, and *Strongyloides stercoralis* were negative. Vitamin deficits and autoantibody battery study were also within normal ranges. Feces samples were negative for helminths. The tuberculin skin test was performed twice with negative results.

A cranial and spinal magnetic resonance imaging demonstrated small demyelinating focal lesions in the subcortical white matter, mainly around the posterior portion of both oval centers, conditioning Wallerian degeneration in the pyramidal tracts bilaterally. The lesions showed no signs of activity.

The cerebrospinal fluid showed lymphocytic pleocytosis. An oligoclonal band study was negative and microscopy did not observe any atypical cells.

On electromyography examination, a severe involvement of pyramidal and somatosensorial tracts for all 4 limbs was diagnosed, suggesting demyelination. No signs of peripheral neuropathy or motor neuron disease were detected.

Furthermore, a skin punch examination of the papular rash of the lower abdomen demonstrated an atypical T phenotype lymphoid infiltrate, suggesting an indolent lymphoma form; however, T-cell receptor γ, T-cell receptor β, and immunoglobulin heavy chain reassortment studies were negative. T CD8 lymphocytes in the epidermis also suggested infective dermatitis. Skin cultures were positive for methicillin-sensitive *Staphylococcus aureus*.

A definitive HAM/TSP diagnosis was made with the information above, plus a positive serology for HTLV (enzyme immunoassay Murex HTLV I+II; DiaSorin, uk. Innogenetics, belgium) and a positive HTLV-1 proviral load in peripheral blood.^12^ The seropositivity for HTLV-1 was confirmed by line immunoassay (INNO-LIA HTLV I/II Score; Innogenetics). Curiously, the line immunoassay also resulted positive for HTLV-2. The partner of the patient also had antibodies against HTLV-1 and HTLV-2 confirmed by line immunoassay.

The HTLV-1 proviral load in the peripheral blood before treatment was 1.767/10.000 peripheral blood mononucleated cell. The proviral load from the cerebrospinal fluid was not performed.

Clinical status was evaluated using the modified Ashworth spasticity scale, spasticity scale-88 score (SPAST-88), SF-36 health survey scale (SF-36), multiple sclerosis walking scale, Instituto de Pesquisa Clínica Evandro Chagas disability score (Instituto de Pesquisa Clínica Evandro Chagas disability score), the Barthel scale, postvoid residual urine volume, nocturia frequency (per night), and a walking test (time to walk 10 m). These tests were performed before treatment initiation and after 62 weeks of treatment. Results are shown in Table 1.

**TABLE 1:**
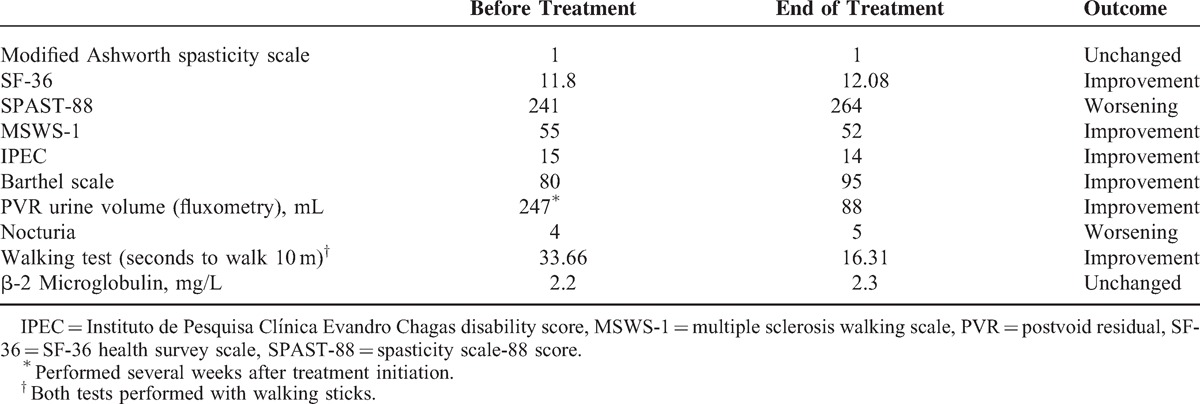
Clinical and Laboratory Outcomes

Cyclosporine was started at 25 mg twice a day and titration was done according to plasma cyclosporine levels. Target plasma levels were 80 to 100 ng/mL, and were mainly achieved with 50 mg twice daily.

Treatment was discontinued after 62 weeks. During this time, the patient was closely monitored with blood tests and physical examination. No side effects or evidence of TLL were reported.

The clinical outcomes and the HTLV-1 proviral load in peripheral blood are depicted in Tables 1 and 2, respectively.

**TABLE 2:**
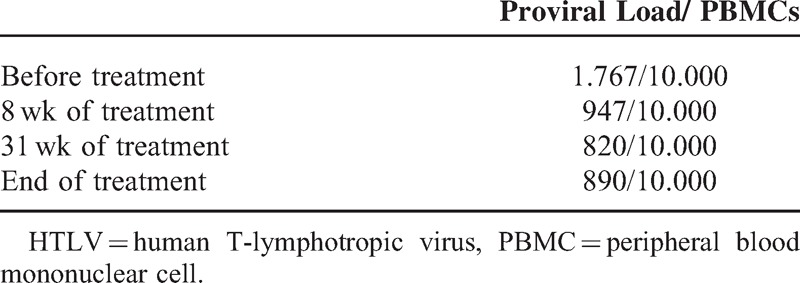
HTLV-1 Proviral Load in Peripheral Blood

## DISCUSSION

Although HAM/TSP was described many years ago, limited efforts have been made to develop new and effective therapies. Different biological approaches have been elaborated to understand the pathogenesis of the disease, and therefore, different treatment options have been tested with inconclusive results, including interferon-α, interferon-γ, antiretroviral therapy, danazol, corticoids, valproic acid, monoclonal antibodies anti-CD25, and others.^9^

Despite the fact that available scientific evidence does not support any treatment, the interferon-α treatment strategy is the most widely studied, and there appear to be benefits when applied in the early or progressive stages of the disease. On the contrary, steroids remain a useful resource for punctual use, but their activity seems temporary and treatment potency is lost with continuous use.

Recently, a proof-of-concept study has supported a potential advantage when using cyclosporine, a steroid-sparing therapy, in the early or progressive phase of the disease.^10^ Our baseline and end-of-treatment outcome criteria were compared after 62 weeks of treatment, in contrast with the 48 weeks of treatment and 72 weeks of follow-up in the proof-of-concept study. Despite these differences, we think our experience strengthens the outcomes obtained by Martin et al.^10^

The IPEC and walking tests were the main monitoring criteria used, as they are stable over time and reproducible because they are rarely modified by mood state or psychiatric conditions. Other tests like SF-36 and SPAST-88 may be highly dependent on mood state, so that a state of depression, usually accompanying the disease, may alter the results. Our patient showed a significant improvement in both the walking and IPEC tests.

Cyclosporine levels were used to adjust the treatment dose. Three out of the 8 cyclosporine levels were in the range between 80 and 100 ng/mL, 1 was above the upper limit, 2 were between 60 and 65 ng/mL, and 2 determinations were <50 ng/mL. Notwithstanding, both determinations <50 ng/mL were the 2 first determinations (performed in the first month) when there was titration of the dose. We can therefore say that cyclosporine levels were within the normal range or above in 50% of the determinations. Treatment was given under directly observed treatment.

HTLV-1 proviral load was only assessed in peripheral blood. Proviral load gradually declined during treatment, as shown in Table 2. How cyclosporine attains this outcome is not well understood.

Although the patient received immunosuppressive therapy for over a year, no sign of hematological disease appeared. However, a concern exists over whether a longer period of treatment may increase the incidence of TLL. More data regarding this aspect are needed.

To summarize, we have reported the case of a patient with an early HAM/TSP treated with cyclosporine, with a satisfactory clinical outcome after 62 weeks of treatment. Cyclosporine seems to be effective in new-onset HAM/TSP or in chronic HAM/TSP that develops a relapse. However, the duration and safety profile of this steroid-sparing therapy remain unknown and should be further investigated.
