# Cyclosporine for the Treatment of HLTV-1-Induced HAM/TSP: An Experience from a Case Report: Erratum

**DOI:** 10.1097/01.md.0000464210.01415.19

**Published:** 2015-02-13

In the article “Cyclosporine for the Treatment of HLTV-1-Induced HAM/TSP: An Experience from a Case Report,^1^ which appeared in Volume 94, Issue 1 of *Medicine*, a word in the title was misspelled. The correct title is “Cyclosporine for the Treatment of HTLV-1-Induced HAM/TSP: An Experience from a Case Report. The article has since been corrected online.
